# Differentiating Left Ventricular Remodeling in Aortic Stenosis From Systemic Hypertension

**DOI:** 10.1161/CIRCIMAGING.123.016489

**Published:** 2024-08-20

**Authors:** Masliza Mahmod, Kenneth Chan, Joao F. Fernandes, Rina Ariga, Betty Raman, Ernesto Zacur, Ho-fon Royce Law, Marzia Rigolli, Jane M. Francis, Sairia Dass, Kevin O’Gallagher, Saul G. Myerson, Theodoros D. Karamitsos, Stefan Neubauer, Pablo Lamata

**Affiliations:** University of Oxford Centre for Clinical Magnetic Resonance Research, Division of Cardiovascular Medicine, Radcliffe Department of Medicine (M.M., K.C., R.A., B.R., M.R., J.M.F., S.D., S.G.M., S.N.), University of Oxford, United Kingdom.; Department of Biomedical Engineering (E.Z.), University of Oxford, United Kingdom.; Department of Biomedical Engineering (J.F.F., H.-F.R.L., P.L.), King’s College of London, United Kingdom.; Department Cardiovascular Medicine (K.O.G.), King’s College of London, United Kingdom.; 1st Department of Cardiology, Aristotle University, Thessaloniki, Greece (T.D.K.).

**Keywords:** aortic valve, aortic valve stenosis, hypertension, hypertrophy, left ventricular, principal component analysis

## Abstract

**BACKGROUND:**

Left ventricular (LV) hypertrophy occurs in both aortic stenosis (AS) and systemic hypertension (HTN) in response to wall stress. However, differentiation of hypertrophy due to these 2 etiologies is lacking. The aim was to study the 3-dimensional geometric remodeling pattern in severe AS pre- and postsurgical aortic valve replacement and to compare with HTN and healthy controls.

**METHODS:**

Ninety-one subjects (36 severe AS, 19 HTN, and 36 healthy controls) underwent cine cardiac magnetic resonance. Cardiac magnetic resonance was repeated 8 months post-aortic valve replacement (n=18). Principal component analysis was performed on the 3-dimensional meshes reconstructed from 109 cardiac magnetic resonance scans of 91 subjects at end-diastole. Principal component analysis modes were compared across experimental groups together with conventional metrics of shape, strain, and scar.

**RESULTS:**

A unique AS signature was identified by wall thickness linked to a LV left-right axis shift and a decrease in short-axis eccentricity. HTN was uniquely linked to increased septal thickness. Combining these 3 features had good discriminative ability between AS and HTN (area under the curve, 0.792). The LV left-right axis shift was not reversible post-aortic valve replacement, did not associate with strain, age, or sex, and was predictive of postoperative LV mass regression (R^2^=0.339, *P*=0.014).

**CONCLUSIONS:**

Unique remodeling signatures might differentiate the etiology of LV hypertrophy. Preliminary findings suggest that LV axis shift is characteristic in AS, is not reversible post-aortic valve replacement, predicts mass regression, and may be interpreted to be an adaptive mechanism.

**CLINICAL PERSPECTIVE**

Persistent pressure overload on the left ventricle in patients with systemic hypertension and aortic stenosis (AS) often leads to left ventricular (LV) hypertrophy, which is known to associate with adverse clinical outcomes. Distinguishing the predominant etiology of LV hypertrophy in patients with coexisting hypertension and AS in the clinical setting could be challenging, and the treatment options for these 2 conditions are different (optimizing pharmacological hypertension treatment and aortic valve intervention, respectively). Using a statistical shape model (SSM) that encodes the detailed 3-dimensional morphology of the LV from cardiac magnetic resonance images, we derived a quantitative measure of each shape signature, which could tease apart the complex remodeling pattern. A higher SSM score in left-to-right axis shift with septal hypertrophy and short-axis eccentricity was observed in remodeling driven by AS, whereas a relatively higher SSM score in septal hypertrophy would suggest a higher equity from hypertension. Furthermore, the SSM signature was detectable in an asymptomatic patient with severe AS and was significantly associated with LV mass regression 8 months after surgical aortic valve replacement. Taken together, the SSM approach to phenotype LV hypertrophy could differentiate remodeling in systemic hypertension and AS, which could guide therapeutic directions and potentially help to identify patients who would derive significant benefits from aortic valve intervention.

**See Editorial by Heydari and Jerosch-Herold**

Aortic stenosis (AS) is a common valvular heart disease, occurring in 2% of the population over 65 years of age, rising to 7% in men over 85 years.^1^ The response of the left ventricle (LV) to AS begins with a compensatory hypertrophic response, which serves to normalize LV wall stress. This is characterized by myocyte hypertrophy, thickened LV wall, and increased LV mass.^2,3^ It ultimately transforms to a maladaptive process, which carries adverse cardiovascular risks, and reversal of this process is accompanied by improvement in outcome.^4–6^ Surgical aortic valve replacement (AVR) and transcatheter AVR are the definitive treatments of symptomatic severe AS, while in asymptomatic severe AS these are recommended if there is LV systolic dysfunction.^4–7^

Systemic hypertension (HTN) presents with a similar pattern of LV remodeling, which is mainly concentric LV hypertrophy, although eccentric remodeling has been described in about a third of cases.^8^ While the increased afterload can affect the myocardium in both HTN and AS, the activation of neurohormonal pathways is an additional mechanism that can affect LV remodeling and performance in hypertension.^9,10^ HTN is also common in patients with AS and can lead to more severe LV remodeling, premature disease progression, and augmented aortic valve calcification.^11–13^ When both conditions are present, it is often difficult to differentiate the etiology of LV hypertrophy in clinical practice. Accurate assessment of LV geometry in AS and HTN could help identify the predominant pathology and therefore guide treatment strategies.

Cardiovascular magnetic resonance (CMR) is an accurate, reproducible, and well-validated assessment of cardiac structure and function, and conventional metrics extracted from this imaging modality are mass, volumes, and function.^14^ Recent advances in computational anatomy tools now enable a much more detailed analysis of the LV remodeling through the construction of statistical shape models (SSM; also referred to as statistical or computational atlases) of the 3-dimensional (3D) geometry of cardiac structures.^15^ The use of these computational atlases has revealed the impact of a premature birth in the adult heart^16^ and has identified a remodeling signature that predicts response to cardiac resynchronization therapy.^17^

Using advanced computational anatomy tools, we sought to test these hypotheses: (1) there is a unique remodeling pattern in AS when compared with hypertension; (2) there is an AS remodeling signature that differentiates between symptomatic and asymptomatic patients; and (3) the remodeling pattern associated with AS recovers after AVR.

## METHODS

An exploratory study on 109 CMR data sets from 91 participants (18 AS subjects had follow-up data after surgical AVR) was conducted to study the end-diastolic LV morphology with robust and reproducible statistical shape models. Anonymized data and materials have been made publicly available at the FigShare repository and can be accessed at 10.6084/m9.figshare.25428604.

### Study Population

Thirty-six severe patients with AS, including 26 symptomatic who had New York Heart Association class ≥2 and/or Canadian Cardiac Society Angina, and 10 asymptomatic, were prospectively recruited from the Oxford University Hospital National Health Service Trust. Severe AS was diagnosed based on the established criteria.^7^ All patients had no evidence of significant coronary artery stenosis as shown by invasive coronary angiography. Patients with AS were included if they had all the criteria of an aortic valve area of ≤1.0 cm^2^, mean pressure drop (PD) of ≥40 mm Hg, maximum jet velocity of ≥4.0 m/s, and absence of other significant valvular pathology based on clinical echocardiogram. Patients were excluded if they had LV ejection fraction <50%, systolic blood pressure (BP) ≥160 mm Hg, and diastolic BP ≥90 mm Hg, contraindications to MR imaging, glomerular filtration rate <60 mL/min, underlying cardiomyopathy such as hypertrophic cardiomyopathy, previous myocardial infarction, coronary revascularization, or previous cardiac surgery. Of the 26 patients with symptomatic AS who underwent AVR, 18 had a follow-up scan 8 months after AVR. Eight patients did not have a follow-up scan due to perioperative death (2 patients), pacemaker implantation (1 patient), lost to follow-up (1 patient), and did not consent for a repeat CMR (4 patients).

For comparison, 19 patients with HTN were recruited. All patients with HTN had a minimum 2-year history of uncontrolled hypertension with ≥2 antihypertensive agents and had ambulatory BP monitoring ≥140/90 mm Hg. In addition, 36 healthy volunteers were recruited as healthy controls; these were asymptomatic, not on medications, had no history of heart disease, diabetes, HTN, or high cholesterol, and had normal physical examination. They were identified from the local population by word of mouth and poster advertisements around hospitals and universities. All subjects gave their informed written consent to participate in the study, which was approved by the Institutional Ethics Committee (Ethics number 07/H0607/66).

### Cardiac Magnetic Resonance and Quantification of Ventricular Volumes, Mass, and Function

**Figure 1.**
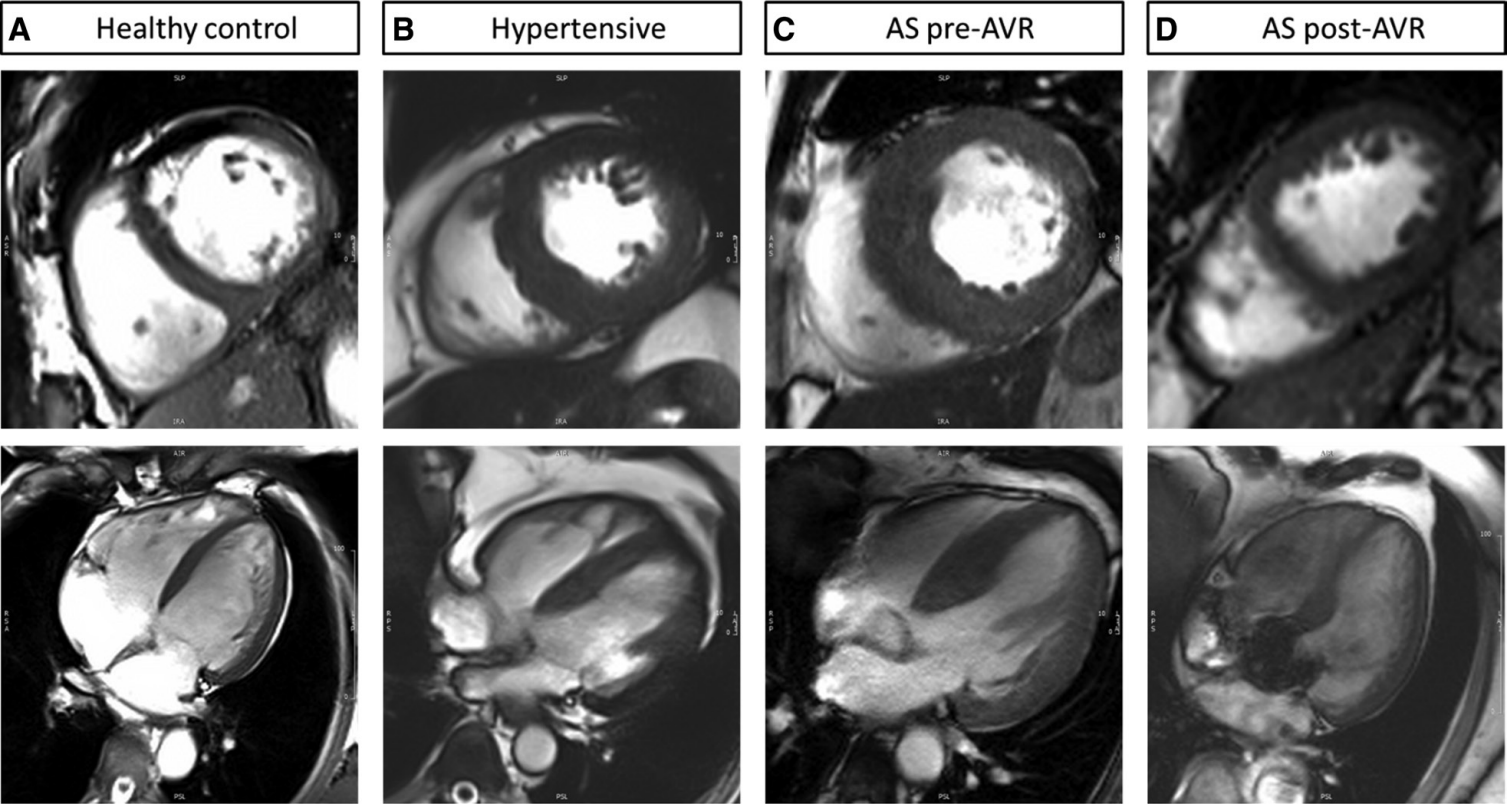
**Representative short-axis and 4-chamber view of cardiac magnetic resonance images. A**, Healthy control; **B**, patient with hypertension; **C**, aortic stenosis pre-aortic valve replacement (AVR); and **D**, post-AVR of the same patient with aortic stenosis (AS).

All subjects underwent CMR scanning on a 3 Tesla MR system (TIM Trio; Siemens Healthcare, Erlangen, Germany). Cine imaging was performed using steady-state, free precession breath-hold in long-axis planes and sequential 7-mm short-axis (SAX) slices from the atrioventricular ring to the apex with a 3-mm gap.^18^ Late gadolinium enhancement images were acquired using standard methods as previously described.^19^ Analysis of cardiac volumes, function, and mass was performed according to standard methods. In the AS cohort, aortic valve area was measured from direct planimetry during aortic valve CMR cine. Late gadolinium enhancement was determined if it was present or not by 2 independent CMR readers.^19,20^ Two-dimensional feature-tracking strain analysis was used to derive peak global longitudinal strain from horizontal long-axis cines, while SAX cines were used to derive circumferential and radial strain. All measurements were performed with CMR42 software (version 4.0; Circle Cardiovascular Imaging). Representative images are illustrated in Figure 1.

### SSM for Assessment of LV Geometry

Creation of an SSM was undertaken using previously published methods.^21,22^ The end-diastolic frame of the SAX cine stack was manually contoured (LV endocardium and epicardium, RV endocardium) with CVI42 (Circle Cardiovascular Imaging). 3D computational meshes were then fully automatically fitted to contours. The LV mesh of each subject was then described with a mesh defined by a set of 3456 nodal variables (or degrees of freedom). See Supplemental Material S1 for a detailed description of the construction of the SSM.

Once the anatomic information has been captured in these meshes, all 109 shapes were prealigned by their center of mass, by setting the vertical direction as the perpendicular to the SAX slice, and by correction of the relative direction aligning the center of mass of LV and RV. The average anatomy was found, and a principal component analysis (PCA), a dimensionality reduction technique, was applied to identify the key “PCA modes of anatomic variation” (ie, the directions of 3D anatomic change that explain the variability of morphologies observed in the 109 shapes). Extreme shapes (±3 SD) of each PCA mode were visually inspected to describe the qualitative shape changes, and the first PCA modes that explained up to 99.5% of shape variability were selected. Each anatomy was now represented as the average anatomy plus the linear combination of the information contained in each PCA mode.

Once the anatomic information is compacted, a supervised learning technique is used to identify the relevant remodeling patterns. The linear combination of the PCA modes that best distinguish between pairs of clinical groups (AS versus control; HTN versus control; AS versus HTN; AS versus AS post-AVR; asymptomatic AS versus symptomatic AS) was identified using linear discriminant analysis (LDA) as in previous studies.^23,24^ Finally, the LDA shape biomarkers are analyzed against clinical parameters using linear regression.

### Statistical Analysis

Clinical baseline characteristics and CMR data were checked for normality using Shapiro-Wilk test. Non-normally distributed variables are presented as median with interquartile range, and compared using nonparametric tests (Mann-Whitney *U* test and Kruskall-Wallis test). Categorical data are presented as frequency with percentage and compared using proportions and χ^2^ test or Fisher’s exact test where appropriate. For characteristics that are significantly different among the 3 groups, post hoc analyses with Bonferroni adjustment were performed to compare subgroups. Comparisons of strain and PCA modes between pairs of experimental groups were performed by an unpaired *t* test. PCA and LDA were performed with MatLab (The Mathworks, Natick, MA). The generalizability of the LDA shape biomarkers to distinguish between pairs of clinical groups was tested by the area under the curve (AUC) in a leave-one-out cross-validation test as described previously.^22^ See Supplemental Material S2 for a detailed justification of this choice.

## RESULTS

### Characteristics of Study Cohorts

There were no significant differences in age, sex, body mass index (BMI), BP, or heart rate between controls and AS. There were no significant differences in sex and BMI between AS and HTN, but HTN subjects were younger (51.4±13.2 versus 67.6±10.2 years) and had higher BP and heart rate (85.9±16.6 versus 66.8±9.7 bpm) than AS (Table 1).

**Table 1.**
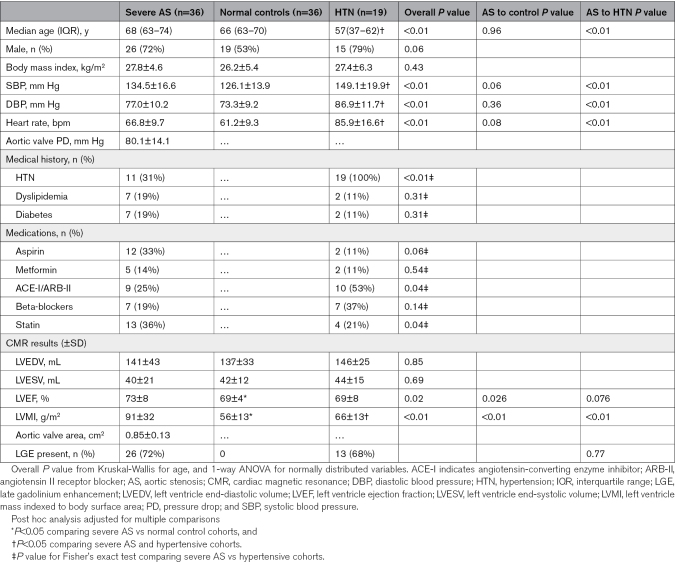
Baseline Characteristics and CMR Results of All Cohorts

Of the 26 patients with symptomatic AS, 18 (69%) had dyspnea, 8 (31%) had angina, and 1 (<1%) had syncope. CMR results revealed that patients with AS had increased LVMI when compared with HTN and control (Table 1). Global LV longitudinal strain was lowest in HTN when compared with AS and controls, while global circumferential strain was lower in AS and HTN when compared with controls (Table 2).

**Table 2.**
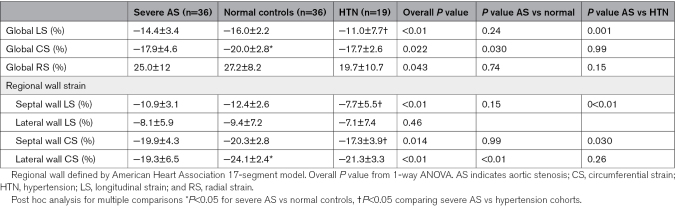
Feature Tracking of Left Ventricular Strain

### Mesh Fitting Accuracy and Pattern of Geometric Shapes

**Figure 2.**
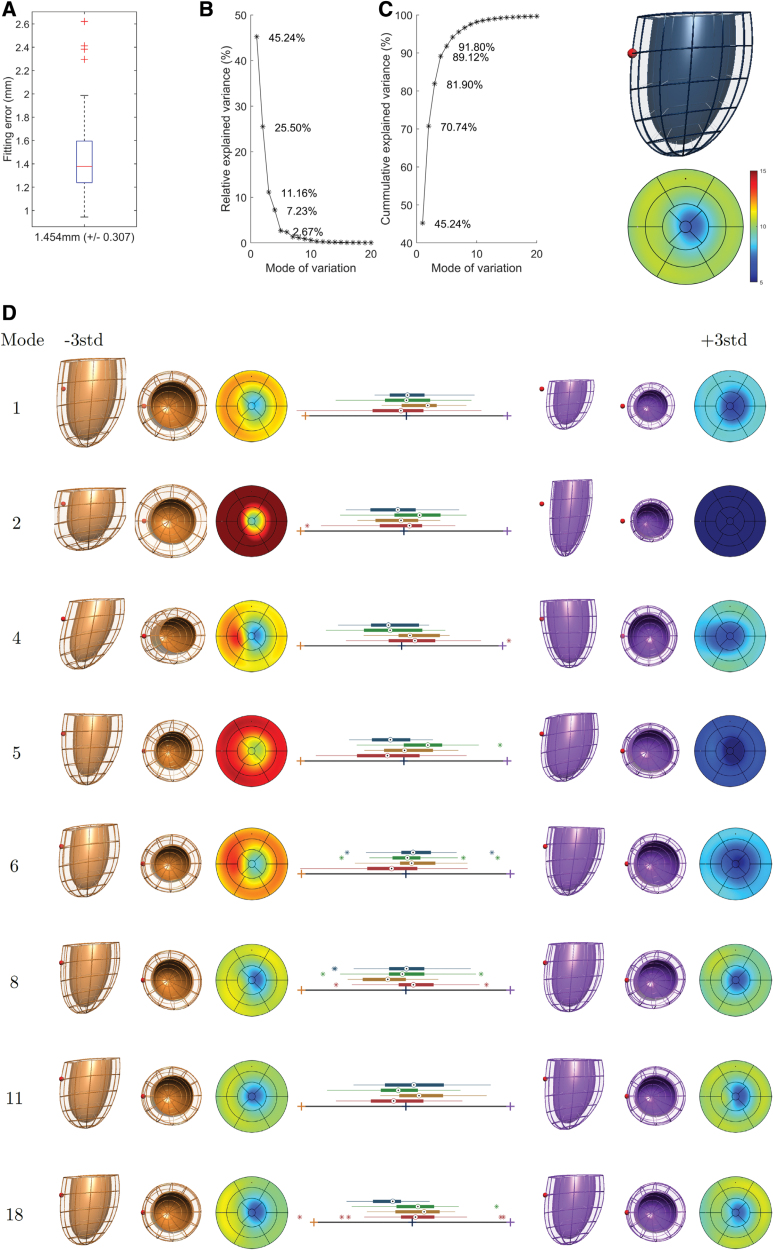
**Statistical shape model of the left ventricle (LV) anatomy of our cohort. A**, Geometric fitting error of the 109 meshes. **B**, Individual and cumulative variance in LV shape explained by the principal component analysis (PCA) modes. **C**, Anterior view of the average 3D shape (red sphere located in septal wall) and its corresponding thickness bullseye plot (in mm). **D**, Extreme shapes (±3 SD) encoded in each PCA mode, with box-plots of the 4 experimental groups (aortic stenosis [AS] in red, n=36; AS post-aortic valve replacement [AVR] in golden, n=18; controls in green, n=36; hypertension [HTN] in blue, n=19).

The LV mesh derived from the contours achieved excellent subvoxel accuracy with an average fitting error of 1.24 mm (Figure 2A). The first 18 PCA modes of variance accounted for 99.56% cumulative variance captured in the 109 meshes (Figure 2B) over the average LV 3D shape illustrated in Figure 2C. The modes that captured differences are illustrated in Figure 2D. See Supplemental Material S3 for a detailed description and interpretation of the first 18 modes of the SSM.

### Comparison of Geometric Changes in AS, HTN, Healthy Controls, and Post-AVR

**Figure 3.**
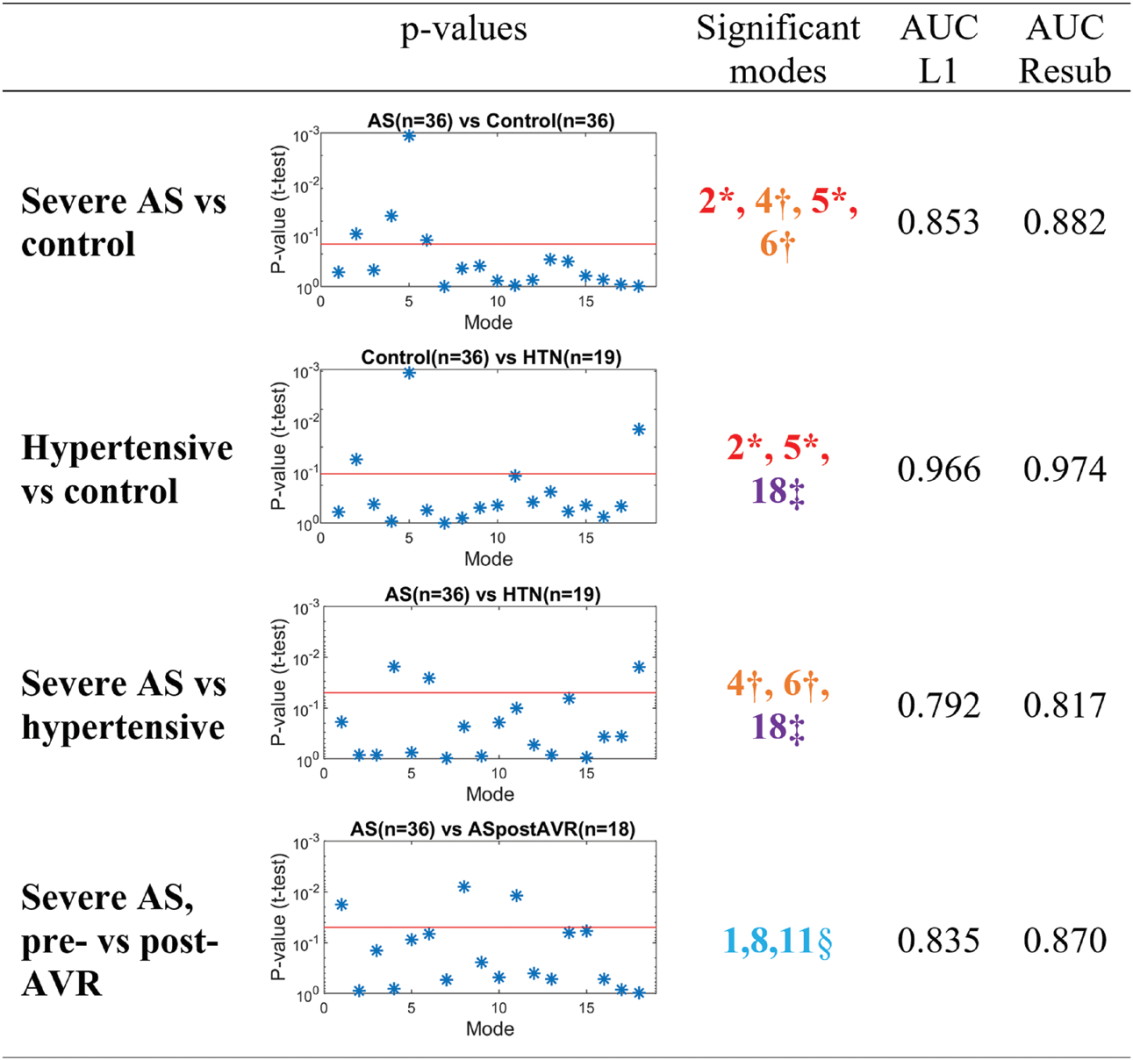
**Identification of principal component analysis (PCA) anatomic modes that are significant (*P*<0.05) in differentiating between experimental groups.** *The signatures of an increased afterload are modes 2 and 5 (common in aortic stenosis [AS] and hypertension [HTN] when compared with controls). †The unique signatures of AS are modes 4 and 6 (both differentiate AS from HTN and controls). ‡The unique signature of HTN is mode 18. §The signatures of correction of AS by AVR are modes 1, 8, and 11. See Figure 2 for an illustration and interpretation of these modes. AUC indicates area under the curve; AVR, aortic valve replacement; L1, leave-one-out cross-validation; PCA, principal component analysis; and Resub, resubstitution.

**Figure 4.**
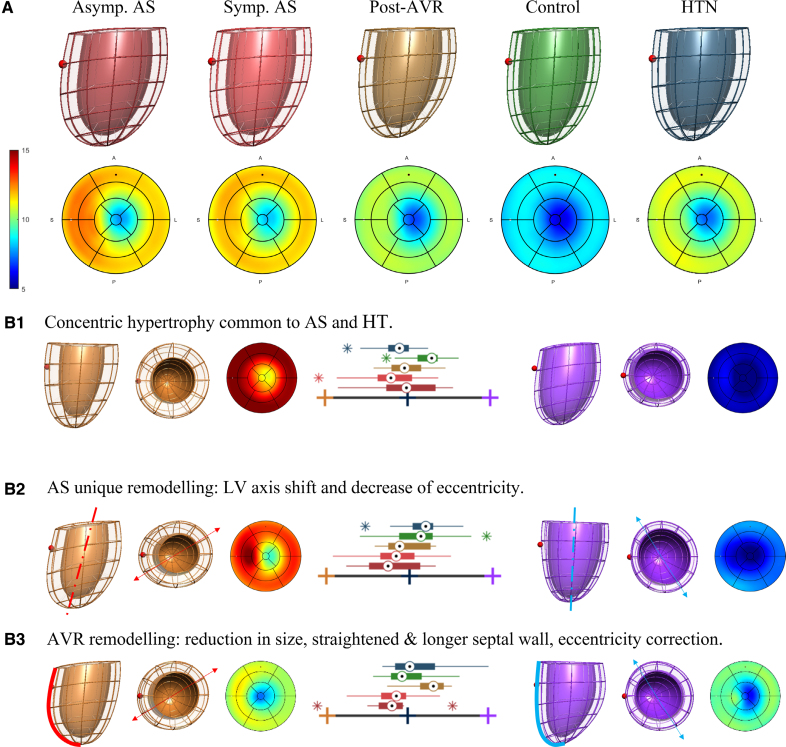
**Average shapes and discriminative axes of remodeling. A**, Average anatomies and corresponding thickness bullseye plot (in mm) of asymptomatic severe aortic stenosis (AS) before aortic valve replacement (AVR; dark red, n=18), symptomatic (light red, n=18), post-AVR (gold, n=18), controls (green, n=36), and hypertension (HTN; blue, n=19). The red sphere indicates the location of the right ventricle. **B1**, The axis that captured the common traits of AS and HTN vs controls (ie, linear discrimination analysis [LDA] from modes 2 and 5), illustrated by the extreme shapes in orange and purple with corresponding extreme box-plot scores at ±3 SD. **B2**, The axis that captured the unique traits of AS vs controls and HTN (ie, LDA from modes 4 and 6). **B3**, The axis that captured the impact of AVR (ie, LDA from modes 1, 5, 6, 8, and 11). LV indicates left ventricle.

Four group comparisons were made. Common and unique discriminative features found are summarized in Figure 3 and illustrated in Figure 4.

The common impact of HTN and AS in LV anatomy was the development of LV hypertrophy, as shown by the combination of PCA mode 2 (reduced LV length and increased LV wall thickness) and PCA mode 5 (concentric hypertrophy). Mode 5 was the feature that best differentiated controls from both AS and HTN.

In addition to these common characteristics, 2 unique PCA modes that differentiated AS from both controls and HTN were found: AS displayed an LV axis shift (apex shifts to the right, mode 4) as a predominant feature, combined with an outward remodeling of the outflow track of mode 6. Both modes also displayed increased wall thickness and a decrease in SAX eccentricity, that is, the ratio between the left-right to the anterior-posterior diameter,^25^ linked to AS. Combining the significant modes 2, 4, 5, and 6, an excellent discriminatory performance was reached between AS and controls (leave-one-out-AUC=0.853).

HTN was also found to have a unique characteristic that differentiated it from the AS and control groups: the presence of a mild septal hypertrophy as captured by PCA mode 18. HTN shape by modes 2, 5, and 18 reached an outstanding discriminative ability between HTN and controls (leave-one-out-AUC=0.966).

The discrimination between AS and HTN was the most challenging task: combination of the significant PCA modes 4, 6, and 18 led to a good performance (leave-one-out-AUC=0.792).

Finally, AVR resulted in a reduction in LV size (mode 1), correction of SAX eccentricity (mode 8) and a longer and straightened septal wall (mode 11), reaching an excellent discriminative ability between AS pre- and post-AVR (leave-one-out-AUC=0.835). Although not reaching statistical significance, AVR showed the tendency of reduction in LV wall thickness, both independent (mode 5) and associated with SAX eccentricity (mode 6). There was no correction for mode 2 (thicker walls and shorter LV) and mode 4 (LV axis shift) detected.

### Comparison of Geometric Changes Between Symptomatic and Asymptomatic AS

No differences were found in any of the 3 axes of remodeling (the concentric remodeling common to AS and HT, the unique AS remodeling, and the AVR remodeling; see Figure 4B1 through 4B3) between the symptomatic and asymptomatic AS subgroups. In the study of the individual PCA modes, the asymptomatic have less concentric hypertrophy accordingly to mode 5 (*P*=0.026) and a tendency of more concentric hypertrophy and increased sphericity accordingly to mode 2 (*P*=0.18), with a net effect of no change in thickness or mass but an increase in sphericity.

### Correlations Between Clinical Parameters Before and After AVR

At baseline before AVR, we investigated the link between LV morphology and LV function. LV thickness and length (modes 1 and 2) had good correlations with global systolic strain (*P*=0.0004, *P*=0.0001, and *P*=0.004 for radial, circumferential, and longitudinal strain, respectively; Figure 3). A linear combination of modes capturing LV axis shift and outflow tract remodeling (modes 3, 4, 6, 7, and 8) was best associated with baseline aortic PD (Figure 3).

We also investigated the ability of anatomic biomarkers to predict the outcomes of AVR. Baseline ventricular thickness, cavity sphericity, and outflow tract remodeling (modes 1, 5, 6, 7, and 8) were best correlated with aortic PD after AVR (*P*=0.002) as well as the absolute PD reduction (*P*<0.001). However, this remodeling pattern was not predictive of the percentage of mass regression at 8-month follow-up (R=0.16, *P*=0.11). Instead, the unique AS signature of LV axis shift (mode 4) correlated with mass regression (R^2^=0.339, *P*=0.014). Besides, mode 4 did not display any association with age, sex, or strain.

## DISCUSSION

We found a unique LV hypertrophy remodeling pattern in severe AS that is distinctive from systemic HTN: severe AS displayed an LV axis shift that is observed in both asymptomatic and symptomatic subgroups. For patients with symptomatic AS who underwent AVR, the LV axis shift did not change significantly in the postoperative follow-up CMR but was associated with a greater degree of LV mass regression, suggesting that the LV axis shift could be an adaptive remodeling pattern in response to the stenotic aortic valve. Our results suggest that a quantitative differentiation of the LV remodeling pattern between AS and HTN is possible with SSM. This would have early clinical translational value as distinguishing the 2 conditions would guide different therapeutic directions (valve intervention versus optimizing hypertension treatment). Supplemental Material S5 illustrates the computation of SSM scores of any new subject.

### A Unique LV Remodeling Differentiating Severe AS and HTN

In patients with both AS and HTN, distinguishing AS from hypertensive LV hypertrophy remains challenging. HTN (ie, reduction in systemic arterial compliance and/or increase in vascular resistance) and AS (ie, proximal increase of resistance to the LV outflow) can cause different types of pressure overload, and we therefore hypothesize that they should also lead to different remodeling patterns beyond the primarily concentric LV hypertrophy.^26^

It has been shown that in patients with AS with concomitant hypertension, symptoms develop early with a relatively larger aortic valve area and lower stroke work loss when compared with patients with isolated AS, which suggests tight control of BP in these patients should be achieved.^26^ Understanding the different contributions of AS and hypertension on ventricular remodeling could facilitate personalized treatment strategies for patients, whether for intervention to relieve valve stenosis or intensify pharmacological treatments for hypertension.

The LV axis shift (ie, mode 4 in Figure 2D) is the most distinctive remodeling pattern found to be specific to AS when compared with the pattern seen in HTN and is a novel feature of our cohort. Similar LV axis shifts have been previously reported to be associated with preterm birth in young adults, that is, PCA mode 4 in Lewandowski et al,^16^ and obesity in children, that is, PCA mode 3 in Marciniak et al.^23^ The early stage of cardiac adaptation in preterm birth and infant obesity suggests that the axis shift is a trait of adaptive remodeling.

In comparison to these previous studies, PCA mode 4 in severe AS also captures a localized increased thickness of the septal wall, indicating the presence of an elevated work/stress in this part of the myocardium linked to the axis shift. Our HTN subjects did also have the feature of thickened septal walls, but milder in magnitude and independent of the LV axis shift (compare mode 18 with mode 4 in Figure 2D). Laplace’s law explains that the flatter the surface, the larger the stress, and it may be the mechanism of the development of these thickened septal walls (since the septum presents a flatter curvature) as in the onset of basal septal hypertrophy in HTN.^27^

The balance of the workload between the septal and lateral walls is a likely associated mechanistic link to explain localized thickening patterns, whereas our patients with HTN displayed an impaired septal longitudinal strain (−7.7±5.5 in HTN versus −12.4±2.6 in controls, see Table 2); in agreement with previous studies,^27^ our patients with AS presented with an impaired lateral wall circumferential strain (−19.3±6.5 in AS versus −24.1±2.4 in controls, see Table 2). The ability to assess regional indexed myocardial work has revealed a progressive imbalance of the distribution of workload in HTN, with a smaller load in the basal regions with respect to the apical regions,^27^ and is thus an opportunity for further research into these mechanistic links.

**Figure 5.**
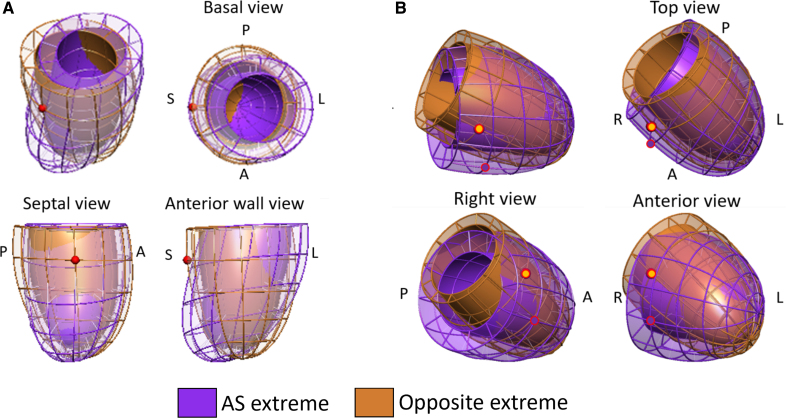
**The impact of aortic stenosis (AS) in left ventricle (LV) morphology in 2 systems of coordinates, illustrated as the overlap between the extreme shapes that maximizes the differences between controls and AS. A**, Results generated in the heart’s local coordinate system (ie, LV shapes aligned by the basal plane and by the direction joining the center of mass of LV and right ventricle [RV]), equivalent to the combination of features illustrated in Figure 2. Note that the red sphere shows the consistent location of the septal wall in both extremes. **B**, Results found in a statistical shape model (SSM) built into the patient’s coordinate system, where the LV shapes are prealigned by their center of mass only. The gold and velvet spheres indicate the different location of the septal wall in each extreme.

This axis shift must be interpreted with respect to the prealignment convention taken when building the statistical shape model, that is, the vertical direction was defined in this and previous studies^16,23^ as perpendicular to the SAX MRI plane. The actual 3D reorientation is illustrated in Figure 5. This is interpreted as the result of the interplay between the elongation of the aorta causing a downward shift of the aortic root, hence increasing the angle between LVOT and the aorta in AS,^28^ and an increase in the local afterload of the stenotic valve (ie, valve acting as a rigid structure in the heart), confined within the fixed pericardial space (which allows the basal plane to be shifted).

We have also identified the known features of ventricular remodeling pattern in AS that share similarities with HTN, such as bulk concentric remodeling (ie, increased ventricular wall thickness captured by modes 2 and 5 in Figure 2D). As expected, the degree of LV hypertrophy was higher in the severe AS group given only mildly elevated BP in the hypertensive group (note also the lower LVMI of the HTN compared with AS, see Table 2). AS and HTN commonly coexist, as shown by a third (31%) of our AS cohort having concomitant HTN, in line with a previous study.^26^ Given the well-controlled BP in the hypertensive AS, it suggests that the LV axis shift was predominantly due to AS.

### Interplay Between LV Anatomy and Function

In a search for further evidence of the adaptive versus maladaptive nature of the changes in LV morphology, we sought to study the interplay between these changes and LV function. The most relevant finding was that only the bulk changes in size (PCA modes 1 and 2 that explain most of the changes in mass, length, volume, or sphericity) correlated with the changes in all 3 global strains. On the contrary, the stenotic burden (ie, the PD) correlated with a certain combination of other morphological features, such as the LV axis shift (see Table 3). LV morphology is accepted to be a contributing factor to the obstruction in hypertrophic cardiomyopathy,^24^ but in AS this causal link is not plausible, and the correlation between LV morphology and stenotic burden is interpreted as additional evidence that the LV adapts to the presence of AS in unique ways beyond hypertrophy.

### Prediction of AVR Outcomes and Understanding the Impact of AVR

In patients with severe symptomatic AS who had AVR, the LV shift remodeling did not change significantly postoperatively after 8 months and was a feature predictive of LV mass regression. We interpret this finding as another supporting evidence to define the LV shift as a trait of an adaptive remodeling, that is, as a compensatory mechanism that heralds the ability to further recover (ie, regress mass) after AVR. It is well established that LV mass regression after aortic valve intervention predicts favorable clinical outcomes such as reduction in heart failure and mortality.^29^

AVR was also associated with a reduction of LV size (mode 1, Figure 1D) when compared with pre-AVR subjects, a different feature compared with the concentric hypertrophy characteristic of HTN and severe AS (modes 2 and 5, Figure 4B1), and a correction of SAX eccentricity and a straightened and longer septal wall (modes 8 and 11, Figure 4B3). This finding suggests that there are 2 different remodeling trajectories: concentric remodeling when the heart gradually adapts to the growing burden caused by a stenotic valve, and the aforementioned characteristics when the heart has a sudden relief of that burden after replacement of the valve. The lack of complete reversal of LV concentric hypertrophy at 8 months may merely indicate incomplete LV recovery, as it has been shown that LV mass regression can occur up to 2 years.^30^ Therefore, future studies evaluating the long-term effect of AVR on LV geometry may be informative.

### Asymptomatic and Symptomatic Severe AS Have Similar Remodeling

The current study found no differences in the main 3 axes of remodeling. There was a tendency for the symptomatic subgroup to display an increased concentric hypertrophy while the asymptomatic subgroup to display a larger axis shift (see Figure 4B1 through 4B3), although these were not statistically significant. The interpretation of the LV axis shift as an adaptive mechanism suggests the hypothesis that the lack of this anatomic trait in severe AS could be detrimental, as it may indicate the inability of the heart to compensate for the extra burden caused by the stenotic valve. The preliminary supportive evidence provided here is the ability of this anatomic trait to predict mass regression, and the SSM might have a role in stratifying asymptomatic patients for early valvular intervention, However, future studies are warranted to contrast this hypothesis and to study the remodeling trajectories in AS (ie, mild and moderate severities) and the interplay between 3D macro-remodeling and micro-remodeling (eg, fibrosis revealed by late gadolinium enhancement) and function, while dissecting sex (eg, male heart develops an accentuated hypertrophic phenotype^31^) and ethnic differences.

### Limitations

There are several limitations of our study. First, the variations in AS shape remodeling are dependent on the total number of hearts analyzed; thus, a larger pool of samples will considerably increase the generalizability of the results and enable the analysis of smaller modes of anatomic variation that encode for subtle hypertrophy patterns. See Supplemental Material S4 for a discussion of the number of PCA modes included.

Second, since our aim was to study patients with hypertension without other comorbidities, our hypertensive patient cohort was understandably younger than patients with AS. To mitigate this limitation, 2 further subanalyses were done: first, a subgroup comparison of age-matched patients (AS group n=30, mean age 67.9±8.4 years; hypertensive group n=9, mean age 62.4±2.7 years; *P*=0.06) was performed and showed minimal impact on the discriminative power between the 2 groups (AUC of 0.719 versus 0.720 with whole cohort). Second, a correlation analysis between age and the PCA modes only revealed modes 8 and 10 as significant (*P*<0.01), modes that are not involved in the differences between AS and HTM groups.

Lastly, the surgical outcome was assessed with follow-up CMR at 8 months. Further prospective study could specifically investigate the long-term prognostic significance of different patterns of ventricular remodeling and differences between AVR and the transcatheter approach.

### Conclusions

Severe AS is characterized by unique LV remodeling patterns when compared with HTN. The LV axis shift is a remodeling trait interpreted as adaptive, that is, associated with mass regression, and that might be a potential marker for personalized risk stratification in the management of AS.

**Table 3.**
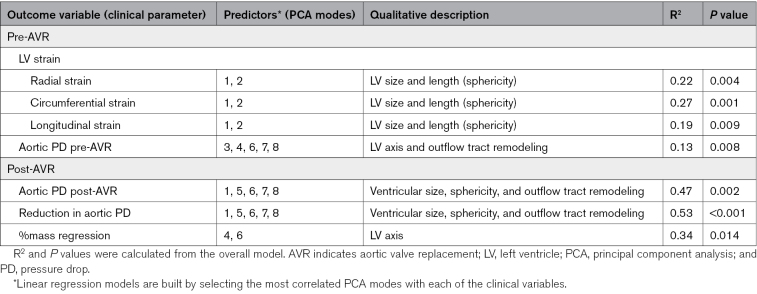
Correlations Between LV Shape and Clinical Parameters Pre- and Post-AVR

## ARTICLE INFORMATION

### Acknowledgments

The authors thank Michelle D’Souza for her contribution in the segmentation task of this work.

### Sources of Funding

This study was supported by a British Heart Foundation Translational Award (TG/17/3/33406); the European Union's Horizon 2020 R&I program under Marie Skłodowska-Curie Actions (764738); a National Institute of Health Research (NIHR) Academic Clinical Fellowship (to Dr Chan); the Wellcome EPSRC Centre for Medical Engineering (WT203148/Z/16/Z); and a Wellcome Trust Senior Research Fellowship (209450/Z/17/Z; to Dr Lamata). Dr Myerson acknowledges funding support from the Oxford NIHR Biomedical Research Centre.

### Disclosures

None.

## Supplemental Material

Supplemental Materials S1–S5
